# Radiography and the Early Diagnosis of Phthisis

**Published:** 1908-05-16

**Authors:** 


### The Hospital

A JOURNAL OF

The Medical Sciences and Hospital Administration.

NEW SERIES. No. 61, VOL. III. [No. 1133, VOL. XLIV.] SATURDAY, MAY 16, 1908.

### RADIOGRAPHY AND THE EARLY DIAGNOSIS OF PHTHISIS.

IF anything were needed to emphasise the curability of pulmonary tuberculosis it might be found in the perseverance with which new means are sought by which the disease can be identified in its earliest phases. As long as a diagnosis of pulmonary tuberculosis was tantamount to a death-sentence nothing was to be gained by hurry in pronouncing the fatal words: indeed the natural tendency was to postpone them to the last possible moment. But when clinical experience and, more particularly, the records of post-mortem rooms gradually established the fact that the spontaneous cure of tuberculous foci was an every-day occurrence, the crying need for an early diagnosis which should anticipate serious damage to the lung was soon appreciated. When, in addition, the discovery of the specific bacillus and the methods of its dissemination became properly understood, a fresh stimulus to early diagnosis was supplied by the knowledge that every case of phthisis is a public menace until the diagnosis is made and the sufferer instructed in his potentialities for harm.

Among the more recent additions to our diagnostic armament in this connection are the Röntgen rays. They have of course been in the field some time, but their usefulness in connection with tuberculosis of the lung has not yet escaped from the sphere of controversy. In the interesting discussion on the subject, conducted lately by the electro-therapeutical section of the Royal Society of Medicine, Dr. Stanley Green affirmed his conviction that unilateral limitation of movement of the diaphragm is the earliest known sign of pulmonary tuberculosis. In support of his contention he quoted the case of patients who had consulted him for hoarseness, without any complaint of lung-symptoms, who nevertheless yielded to radiographic examination definite evidence of tubercular mischief in the lung. As a corollary he remarked that when physical examination has revealed evidences of the disease, but limits it to one lung only, radiography will often demonstrate early lesions in the other lung. He suggested further that the extent of diaphragmatic limitation, together with the depth of the shadows cast by the affected areas of the lung, gives some prognostic indications of importance. Dr. Green's claims were made dogmatically with the avowed intention of provoking discussion, though the speaker insisted that the Röntgen-ray examination must be considered supplemental to the other methods of diagnosis at our disposal, and in no sense as a substitute for them. The most important contribution to the discussion which followed was made by Dr. Lees. He observed that the illustrative skiagrams which had been shown to the meeting appeared to be drawn from cases so advanced that careful percussion would certainly have revealed the disease. He stated that as a result of observations made at St. Mary's Hospital he and his co-adjutor, Dr. Simmons had come to the conclusion that the $x$-rays failed to show any distinct shadow in cases where the loss of resonance was quite definite on careful percussion. He proceeded to define what in his opinion constituted "careful percussion." It must, he said, be very light, and digital only, instrumental aid being fatal to success. The percussed phalanx must be firmly pressed on the point to be percussed, the rest of that finger and the rest of that hand being kept entirely away from the chest wall. Such percussion is capable of detecting stages of pulmonary tuberculosis which are quite early, and which are undiscernible by the $x$-rays. But it is essential that attention should be concentrated upon those areas of the lung in which, as has long since been pointed out by Dr. Kingston Fowler, the very earliest deposits are found. In the upper lobe there are two such areas situated some two inches below the summit of the lung in life: of these areas one is accessible through the first intercostal space close to the manubrium sterni, the other in the same space, but at its outermost part. Behind, the level of these areas corresponds with that of the first dorsal vertebral spine. In the lower lobes the earliest deposits occur at a similar distance below the apex of the lobe, at the level of the inner end of the spine of the scapula.

It is clear that in such a matter the personal equation is the determining factor in the conclusion. In the exercise of the four ordinary means of physical examination the good and the bad examiner are as the poles asunder, and generalisations upon physical examination as a fixed quantity, so to speak, are quite idle. Particularly is this the case when, as is claimed by Dr. Lees, the vital item in the examination is percussion. Accurate and delicate percussion, by which we mean delicacy of touch and accuracy of interpretation, requires a degree of skill and sensibility which some men can readily command and others can never acquire. It is not, therefore, a surprise to meet contradictory opinions upon the less gross lung-lesions; and the fact that the *x*-rays can tell us more than examiner A is no evidence that examiner B would yield them the same proud position. Dr. Green quotes a victory of radiography over three medical men who mistook a case of pyopneumothorax for bronchitis. Yet it is hard to believe that an expert examiner of the lungs could have been guilty of what seems worthy to be termed a careless blunder. On the other hand it is equally probable that the expert radiographer will detect slight abnormalities which remain hidden from his less endowed brother. Excellence of apparatus, technical skill, and cleverness of interpretation cannot fail to influence the results obtained, and this fact again precludes dogmatic assertions on the precedence of one or other method of examination. Without a formal trial of strength between admitted leaders in the two branches of examination this question is never likely to be laid to rest.

